# Prions: The New Biology of Proteins

**DOI:** 10.3201/eid1306.070311

**Published:** 2007-06

**Authors:** Ermias D. Belay

**Affiliations:** * Centers for Disease Control and Prevention, Atlanta, Georgia, USA

Prions are believed to be the causative agents of a group of rapidly progressive neurodegenerative diseases called transmissible spongiform encephalopathies, or prion diseases. They are infectious isoforms of a host-encoded cellular protein known as the prion protein. Prion diseases affect humans and animals and are uniformly fatal. The most common prion disease in humans is Creutzfeldt-Jakob disease (CJD), which occurs as a sporadic disease in most patients and as a familial or iatrogenic disease in some patients. Whether prions are infectious proteins that act alone to cause prion diseases remains a matter of scientific debate. However, mounting experimental evidence and lack of a plausible alternative explanation for the occurrence of prion diseases as both infectious and inherited has led to the widespread acceptance of the prion hypothesis.

Interest in prion disease research dramatically increased after the identification in the 1980s of a large international outbreak of bovine spongiform encephalopathy (BSE, also known as mad cow disease) in cattle and after accumulating scientific evidence indicated the zoonotic transmission of BSE to humans causing variant CJD. In recent years, secondary bloodborne transmission of variant CJD has been reported in the United Kingdom.

Prions: The New Biology of Proteins describes the current state of knowledge about the enigmatic world of prion diseases. The book is organized into 12 mostly brief chapters, which nicely summarize the various types of prion diseases and the challenges associated with their diagnosis and treatment. These sections review the biology of prions, the underlying hypotheses for prion replication, and the biochemical basis for strain diversity. Chapters 2 through 5 describe the various characteristic features of prions, including the historical evolution of the prion hypothesis, a detailed description of the possible mechanisms by which the normal prion protein is converted into the pathogenic form, and the cellular biology and putative functions of the normal prion protein. The author’s lucid descriptions of the various topics are supported by diagrams and key references. Subsequent chapters describe prion disease laboratory diagnostic tools that are available or under development. Chapter 9 succinctly summarizes the most likely target sites, from the formation of the infectious agent to its effects on neurodegeneration, which can be exploited for likely therapeutic development. The same chapter describes the various antiprion compounds that have been or are being tested as therapeutic interventions for prion diseases.

The book is unusual because its entire content was exclusively authored by 1 person, resulting in a paucity of in-depth information in some areas, which may have been provided by multiple authors. However, all things considered, the book can be a valuable resource for scientists beginning to understand the world of prion diseases, the underlying biochemical mechanism of disease occurrence, and the challenges associated with the diagnosis and treatment of prion diseases.

